# A systematic review and meta-analysis of prevalence of insomnia in the third trimester of pregnancy

**DOI:** 10.1186/s12884-021-03755-z

**Published:** 2021-04-09

**Authors:** Nader Salari, Niloofar Darvishi, Behnam Khaledi-Paveh, Aliakbar Vaisi-Raygani, Rostam Jalali, Alireza Daneshkhah, Yalda Bartina, Masoud Mohammadi

**Affiliations:** 1 Department of Biostatistics, School of Health, Kermanshah University of Medical Sciences, Kermanshah, Iran; 2 Sleep Disorders Research Center, Kermanshah University of Medical Sciences, Kermanshah, Iran; 3 Student Research Committee, Kermanshah University of Medical Sciences, Kermanshah, Iran; 4 Department of Nursing, School of Nursing and Midwifery, Kermanshah University of Medical Sciences, Kermanshah, Iran; 5 School of Computing, Electronics and Maths, Coventry University, London, UK; 6 Department of Translation Studies, Faculty of Literature, Istanbul University, Istanbul, Turkey

**Keywords:** Insomnia, Pregnancy, Sleep disorder

## Abstract

**Background:**

Sleep disorders, which are among the foremost important medical care issues, are prevalent in pregnancy. The present study is a meta-analysis of the prevalence of insomnia in the third trimester of pregnancy. This study aims to systematically review the overall prevalence of insomnia in the third trimester of pregnancy through conducting a meta-analysis.

**Method:**

The literature used in this meta-analysis for the topic discussed above were obtained through searching several databases, including SID, MagIran, IranDoc, Scopus, Embase, Web of Science (WoS), PubMed Science Direct and Google Scholar databases without time limitation until December 2020. Articles developed based on cross-sectional studies were included in the study. The heterogeneity of studies was investigated using the I^2^ index. Also, the possible effects of heterogeneity in the studied studies are investigated using meta-regression analysis.

**Result:**

In 10 articles and 8798 participants aged between11–40, the overall prevalence of insomnia in the third trimester of pregnancy based on meta-analysis was 42.4% (95% CI: 32.9–52.5%). It was reported that as the sample size increases, the prevalence of insomnia in the third trimester of pregnancy increases. Conversely, as the year of research increases, the prevalence of insomnia in the third trimester of pregnancy decreases. Both of these differences were statistically significant (*P* < 0.05).

**Conclusion:**

Insomnia was highly prevalent in the last trimester of pregnancy. Sleep disorders are neglected among pregnant women, and they are considered natural. While sleep disturbances can cause mental and physical problems in pregnant women, they can consequently cause problems for the fetus. As a result, maintaining the physical and mental health of pregnant mothers is very important. It is thus recommended that in addition to having regular visits during pregnancy, pregnant women should also be continuously monitored for sleep-related disorders.

## Background

Sleep is an essential physiological need for human beings, and it is also critical for their physical and physiological health [1]. Sleep disorders are considered to be the foremost important issues in medical care. They are generally classified as disturbed quality, poor sleep continuity, Restless Legs Syndrome, sleep disorder and sleep respiratory disorder [2]. Insomnia is a patient-reported compliant defined as difficulty in falling asleep or maintaining sleep, i.e., frequent awakening, difficulty returning to sleep after awakening, or awakening too early with inability to return to sleep [3]. Insomnia is the most common sleep complaint affecting 10–13% population chronically, and up to 35% of the population is experiencing some symptoms of insomnia [4]. A considerable quantity of studies reported that women complained more often of insomnia than men [5]. Pathophysiology and the time insomnia starts is not always clear [6]. Insomnia is typically diagnosed by recording nightly bedtime routine [7].

Pregnancy is one of the most crucial and critical stages in a woman’s life. It brings tremendous changes in women’s physiological, psychological and social life [1, 8]. Around 2/3 of pregnant women consider their sleep pattern has become abnormal [9]. Depending on the women’s body position and increase in their abdomen size, women can experience insomnia during pregnancy [10]. The association of sleep abnormality and pregnancy complications is biologically plausible [9]. Poor health outcomes resultinging from biological, and secretion modifications throughout pregnancy might be associated with sleep disturbances [2]. Sleep disturbances, which are common and prevalent in pregnancy, increase as pregnancy progresses [11]. In pregnancy, sleep changes with raised demand within the first trimester and additional difficulties within the last trimester [12]. In a study, Neau et al. (2009) reported that sleep alterations are common during pregnancy, yet the frequency of such alterations relies heavily on the trimester of pregnancy. Furthermore, Neau et al. [2009] maintain that more than 90% of patients involved in the study asserted that the quality of their sleep was good before pregnancy, but they experienced sleeping difficulty more and more as pregnancy proceeded [13].

A decrease in sleep quality and an increase in total sleep time have been frequently reported by pregnant women [2, 9]. Insomnia in gestation is also caused by several reasons like physical illness, secretion changes, and fetus growth [1]. Insomnia causes deterioration in the quality of life; therefore, it becomes a significant problem throughout gestation for maternal and fetus health [1].

Considering the health benefits of good sleep for pregnant women, it is crucial to find ways to tackle the increasing prevalence of insomnia during the pregnancy and improve the sleep quality of this population [14]. The present study is a meta-analysis of the prevalence of insomnia in the third trimester of pregnancy.

## Methods

### Search strategy

This study is a systematic review and meta-analysis based on the findings of previous studies conducted on the prevalence of insomnia in the third trimester of pregnancy, including articles published in domestic and foreign journals obtained by searching at Scopus, Embase, Science Direct, Web of Science (WoS), SID, Mag Iran, Medline (PubMed), IranDoc and Google Scholar until December 2020.

The search process was performed in Persian and English databases using keywords, including disorders of initiating and maintaining sleep, insomnia, pregnancy and third trimester. The AND/OR operators were also used in this study to provide more comprehensive access to all articles. Therefore, the AND/OR operator was used to check the disorder’s common names by matching words in the MeSH browser.

PubMed search strategy: (((((Sleep [Title/Abstract]) OR Sleep Habits [Title/Abstract]) AND Insomnia [Title/Abstract]) OR Sleep Initiation and Maintenance Disorders OR Sleep Habits [Title/Abstract]) OR Chronic Insomnia [Title/Abstract]) OR Disorders of Initiating and Maintaining Sleep [Title/Abstract]) OR DIMS [Title/Abstract]) OR Nonorganic Insomnia [Title/Abstract]) OR Primary Insomnia [Title/Abstract]) OR Psychophysiological Insomnia [Title/Abstract]) OR Sleeplessness [Title/Abstract]) OR Insomnia Disorder [Title/Abstract]) AND Pregnancy [Title/Abstract]) OR Pseudopregnancy [Title/Abstract]) OR Pregnant Women [Title/Abstract]) AND Third Trimester of Pregnancy [Title/Abstract]) OR Pregnancy Trimester, Third [Title/Abstract]) OR Last Trimester [Title/Abstract]))))).

### Inclusion and exclusion criteria

The studies’ inclusion criteria include 1- Cross-sectional studies, 2- population-based study, 3-studies that have examined insomnia in the third trimester of pregnancy, 4- analytical descriptive studies (non-interventional studies), and 5- studies in the English language.

Exclusion criteria of the studies include 1- case-control studies, 2- case report, 3- interventional studies, 4-letter to the editor, 5- studies with no full-text availability 6- studies not related to the subject, 7- studies without sufficient data, 8- repetition of studies, and 9- review studies.

### Criteria for selection and evaluation of articles and quality assessment

In order to maximize the comprehensiveness of the search, the list of sources used in all related articles found in the above search was manually reviewed. Initially, studies that were repeated in various databases searched were removed from this study. Then, a list of all the remaining articles’ titles was prepared to get qualified articles by evaluating the articles in this list.

In the first stage, duplicate publication and multiple publications from the same population were removed using EndNore citation management software (version X7, for Windows, Thomson Reuters).

The title and abstract of the remaining articles were carefully studied in the next step, and unrelated articles were removed according to the inclusion and exclusion criteria. In the third stage, i.e. the evaluation of the competence of the studies, the full text of the possible related articles remaining from the screening stage was examined based on the inclusion and exclusion criteria, and unrelated studies were also removed in this stage. To prevent bias, all sources of resource review and data extraction were performed by two researchers independently. If the articles were not included, the reason for deleting them was mentioned. In cases where there was a disagreement between two researchers, a third person would review the article.

In the final stage, the quality of the studies was examined, and the STROBE checklist was used to review the studies. This checklist contains 22 sections, 18 of which are general and practical for all observational studies, including cohort, case study, and cross-sectional ones. Accordingly, the maximum quality score of 32 was considered, and papers with a score of less than 14 were considered to have low quality, and they were thus excluded from the study.

### Statistical analysis

In each study, the prevalence of insomnia in the third trimester of pregnancy was obtained. The heterogeneity of the studies was evaluated using the I^2^ test. Generally, heterogeneity is classified into three categories: heterogeneity> 25% (low heterogeneity), 25–75% (average heterogeneity), and 75% > higher (high heterogeneity). In order to review the analysis of the studies, it was decided based on the results of heterogeneity. If the heterogeneity was less than 20, the method of fixed effects was used, and if it was higher, the method of random effects was used. Egger’s test was used to statistically analyze the publication bias and the significance level of 0.05. The probability of publication bias in results was shown using the funnel plot. Also, the possible effects of heterogeneity in the studied studies were investigated using meta-regression analysis. The data were analyzed using the Comprehensive Meta-Analysis (Biostat, Englewood, NJ, USA Version 3).

## Results

**Fig. 1:**
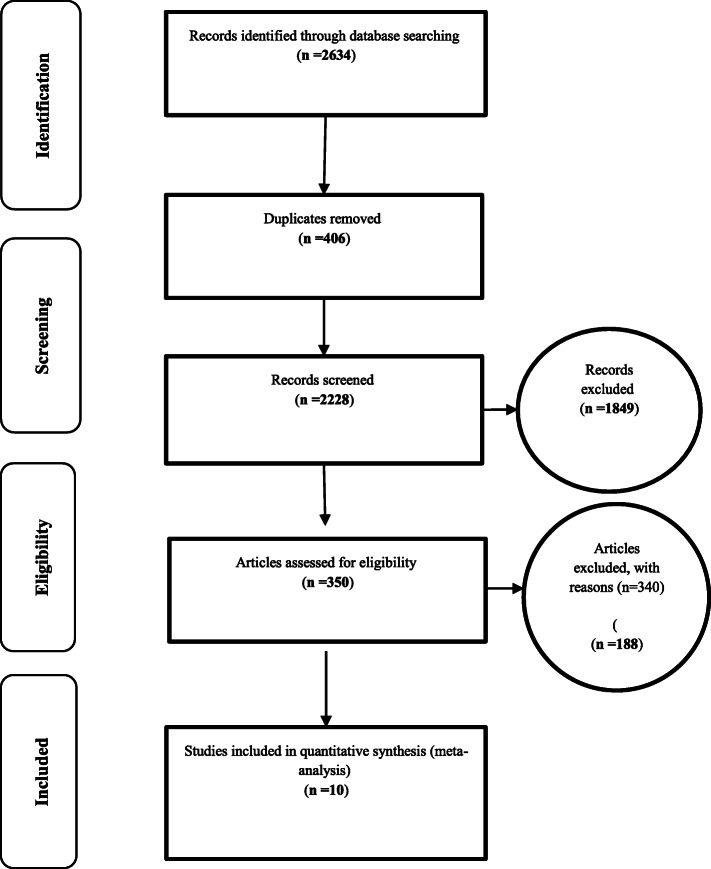
the flowchart the process of evaluating and selecting studies based on the four-step Prisma process (PRISMA 2009)

**Fig. 2:**
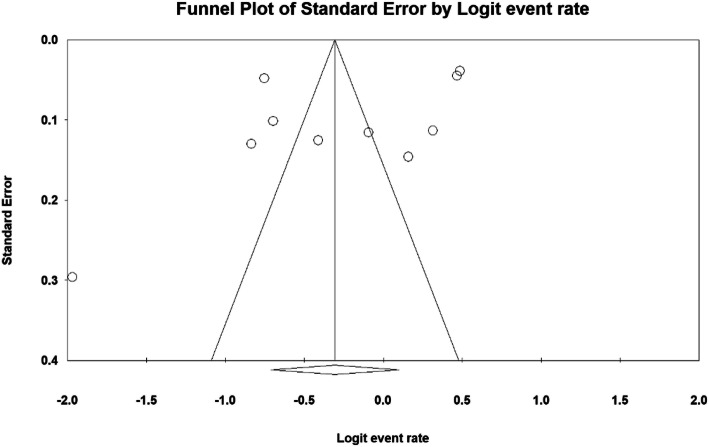
Funnel plot and review of publication bias for the results of the prevalence of insomnia in third trimester of pregnancy

Based on studies on the prevalence of insomnia in the third trimester of pregnancy that included articles published in domestic or foreign journals (Fig. 1), articles that met the initial inclusion criteria were included in the study. In the second step, based on the primary assessments and exclusion of 406 duplicate ones, a total of 2228 articles remained. A further 350 articles were eliminated as they were unrelated to the subject of the study and 18 articles in a secondary assessment due to the lack of access to their abstract and full text as well as their poor quality. Therefore eventually 10 articles were entered in the meta-analysis process (Table 1). The heterogeneity of the studies was evaluated using the I^2^ test that its value was 98.6%, thus the random-effects model was used to combine the results of the studies. The publication bias was not statistically significant (*P* = 0.371) (Fig. 2).

**Table 1:** Specifications and information extracted from studies entered into the meta-analysis stage

Row	Author	Publication year	Area	Participants’ Age	Sample size	Prevalence	Assessing and defining sleep disorders
1	Cai, X. H [15].	2013	China	–	1993	32.01%	Sleep status of the married women was conducted using the Berlin Questionnaire. They adapted a few other items according to the characteristics of the pregnant women in their study.
2	Dørheim, S. K [12]	2012	Norway	30.9(±4.8)	2816	61.9%	Insomnia was measured by the Bergen Insomnia Scale.
3	Dørheim, S. K [10]	2014	Norway	31.5(±4.7; range 17.4–45.7)	2088	61.6%	The Bergen Insomnia Scale (BIS) was used to measure insomnia
4	Facco, F. L. [9]	2010	America	29.7 (±5.5)	189	54.3%	The survey was composed of the following validated sleep questionnaires: Berlin Questionnaire for Sleep Disordered Breathing, Epworth Sleepiness Scale, National Institutes of Health/International Restless Legs Syndrome Question Set, Women’s Health Initiative Insomnia Rating Scale, and the Pittsburgh Sleep Quality Index.
5	Kizilirmak, A [1].	2012	Turkey	25.2 ± 5.49	281	30.24%	developed Women’s Health Initiative Insomnia Rating Scale (WHIIRS): The scale is a 5-point Likert type in which the first 4 questions aim to identify the beginning of insomnia, sleep-maintenance insomnia, and the state of waking up early in the mornings, while the last question is associated with quality of sleep. “0” score indicates that there is no problem in relation to insomnia, while “4” indicates that there are problems in relation to insomnia for 5 or more times in a week. The highest score obtained from the scale demonstrated the highest levels of insomnia symptoms.
6	Khazaie, H [2].	2013	Iran	25.3 ± 5.5	106	12.3%	Global sleep assessment questionnaire (GSAQ)
7	Lopes, E. A [16].	2004	Brazil	11–40	300	39%	The survey was composed by brief clinical interview based on directed questions. An anamnesis was performed according to the following questions, always considering the PG state: 1) Do you have any difficulty to fall asleep when you lie down in bed? 2) Do you wake up too early in the morning (earlier than you are supposed to)? 3) If you wake up during the night, do you find it difficulty to sleep again? 4) Has anyone ever told you that you snore? 5) Has anybody ever said that you have difficulty in breathing during the night (like stop breathing)? 6) Do you suddenly fall asleep during the day or in the middle of some kind of activity? 7) Do you fall asleep anywhere (as on buses, in the classroom, at work or while driving)? 8) Do you feel sleepy during the day? 9) Have you been taking naps during the day? 10) Do you wake up with the baby movements? 11) Do you wake up because of abdominal pains or contractions? 12) Do you wake up due to dreams or nightmares involving the baby or to childbirth? 13) Do you wake up with heartburn?
8	Wolynczyk-Gmaj, D [17].	2017	Poland	30.6 ± 5	266	39.8%	The assessment of variables was performed using the Athens Insomnia Scale (AIS), Beck Depression Inventory (BDI), Regestein Hyperarousal Scale (HS), Epworth Sleepiness Scale (ESS), General Practice Physical Activity Questionnaire, and a semi-structured interview about different sleep disorders
9	Bondad,R [18].	2004	Iran	12.87 ± 4.86	320	57.81%	Check the sleep pattern test (Sleep-log)
10	Okun, M. L [19]	2018	America	28.8 ± 6.3	439	33.25%	The ISQ instrument designed to identify insomnia. The ISQ is a 13-item self-report instrument which questions are based on DSM-IV criteria for primary insomnia and are consistent with the American Academy of Sleep Medicine’s (AASM) Research Diagnostic Criteria (RDC).

**Fig. 3:**
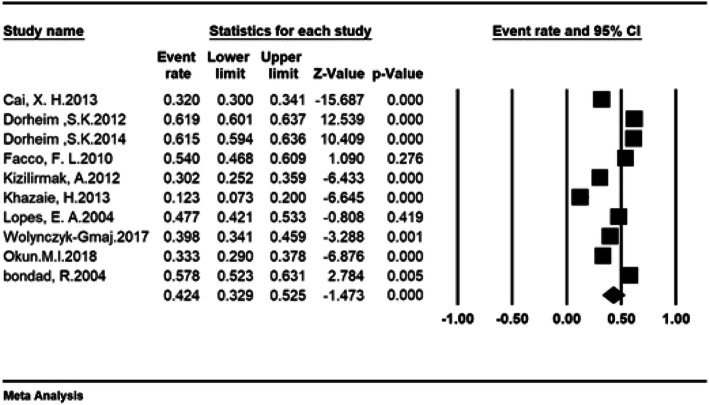
Forrest Plot and Determination overall prevalence of insomnia in third trimester of pregnancy based on the random effects model

**Fig. 4:**
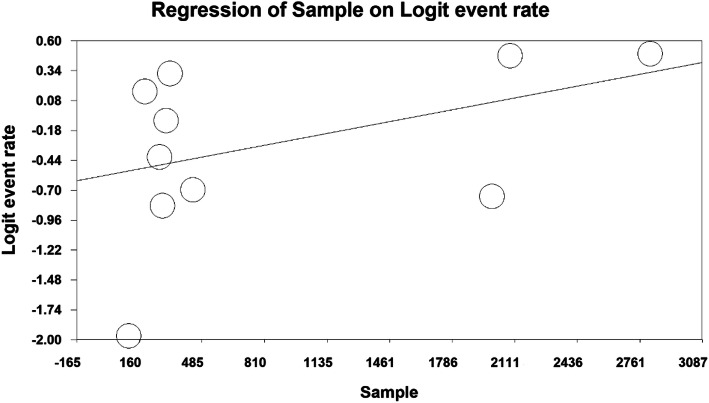
The effect of sample size of studies on the prevalence of insomnia in the third trimester of pregnancy based on meta-regression analysis

**Fig. 5:**
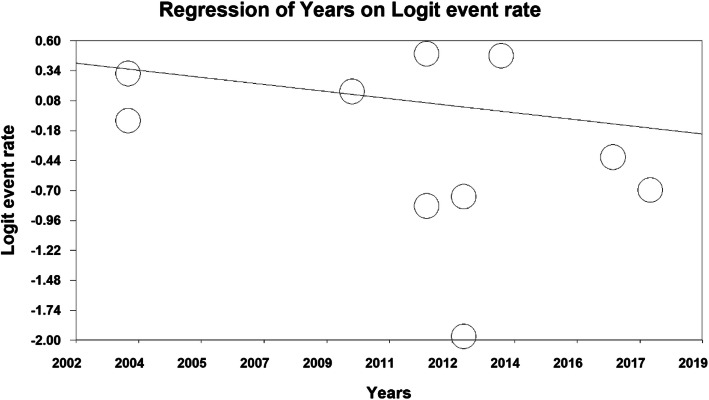
The effect of publication year of studies on the prevalence of insomnia in the third trimester of pregnancy based on meta-regression analysis

The total number of participants in this study was 8798 individuals aged between 11 and 40 years old, and the overall prevalence of insomnia in the third trimester of pregnancy was 42.4% (95% CI: 32.9–52.5%) (Fig. 3). As shown in Fig. 4, as the sample size increases, the prevalence of insomnia increases in the third trimester, which is statistically significant (*P* < 0.05). In Fig. 5, it is also shown that with an increase in the number of years of research, the prevalence of insomnia in the third trimester was reduced, and this was also statistically significant (*P* < 0.05).

## Discussion

This study aimed to determine the prevalence of insomnia during the third trimester of pregnancy. Our systematic review of published epidemiologic studies confirmed a 42.4% prevalence of insomnia in the third trimester of pregnancy. It is the first meta-analysis conducted on the prevalence of insomnia in late pregnancy to the best of our knowledge. In total, we have used ten studies in this meta-analysis to address the purposes of this study.

Pregnant women often experience insomnia. The prevalence of this complication in early pregnancy is between 12 to 38%, which increases to 60% in the third trimester [20]. According to the results of the study by Osnes,. et al. (2020), 60% of the study population has experienced mid-pregnancy insomnia and 55% of these individuals have experienced postpartum insomnia [20]. In a meta-analysis conducted in 2020, the overall prevalence of insomnia symptoms in the study population was 38.2%. According to a study by Felder et al. (2020), approximately 40% of 117 individuals in the study population experienced moderate to severe insomnia symptoms. According to the results of this study, the prevalence of insomnia did not differ in different trimesters of pregnancy, but this complication was more common in women with children compared to women who did not have children [21].

Natural biological changes may play a vital role in the development of insomnia in pregnant women. Women who are prone to insomnia may, for example, have more difficulty in falling asleep after waking up at the midnight due to stressors related to pregnancy [22]. Various factors might be associated with insomnia in pregnancy of that age of pregnant woman, gestational age, pregnancy trimester, symptoms of anxiety and depression, level of education, and personality traits are among these factors [22, 23]. Mid-pregnancy insomnia was associated with the occurrence of depression but was not significantly associated with postpartum depression [20]. Previous background of insomnia is the most important factor in developing this complication during pregnancy [24].

Sleep alterations and daytime sleepiness are commonly reported during pregnancy, but their frequency depends on the trimester of pregnancy [13].

As the pregnancy progresses, the rate of insomnia increases and then decreases significantly after delivery [24]. Many studies have reported that sleep quality deteriorates during pregnancy and is often most disturbed in the third trimester [22]. According to a study by Wolynczyk-Gmaj et al. (2017), the prevalence of insomnia in the third trimester of pregnancy was reported as 39.8%. Roman-Galvez et al. (2018), reported that the prevalence of insomnia in the first, second and third trimesters was 44.2, 46.3, and 63.7%, respectively. According to this study, obesity of pregnant woman, occupational status of pregnant women and previous background of insomnia were introduced as factors related to the high prevalence of insomnia during pregnancy [24].

In a similar study conducted in Poland and reported in Wolynczyk et al. (2017), the prevalence of insomnia in the third trimester of pregnancy was reported to be 39.8% [17]. In another study conducted in Iran, the prevalence of insomnia within the third trimester of gestation was reported to be 57.81%. This study presented insomnia as the most common disorder within the third trimester of pregnancy [18]. Another study in Norway reported an incidence of 61.9% in the third trimester of pregnancy [12]. Likewise, in another study by the same author two years later showed that the incidence of insomnia was 61.6% [23].

Considering the results of the present study obtained from the examination of 8798 individuals with the age range of 11–40, the overall prevalence of insomnia in the third trimester of pregnancy was 42.4% that is based on the meta-analysis. Moreover, the results obtained from meta-regression reported that the prevalence of insomnia in the third trimester of pregnancy increases with increased sample size and reduces as the number of years increases that were both statistically significant.

### Limitations

The most important limitations of the present study were related to the inaccessibility of the full-text of some retrieved studies and the lack of information required in some studies.

## Conclusions

Sleep disturbances can cause mental and physical problems in pregnant women as well as problems for the fetus. As a result, maintaining the physical and mental health of pregnant mothers is of great importance. It is thus recommended that in addition to having regular visits during pregnancy, pregnant women should also be continuously monitored for sleep-related disorders. Appropriate counselling programs to prevent and treat sleep disorders should be then provided. This will take a step towards ensuring the health of pregnant mothers, their babies, and ultimately, the health of the whole community.

## Data Availability

Datasets are available through the corresponding author upon reasonable request.

## Abbreviations

SID: Scientific Information Database
WoS: Web of Science
PRISMA: Preferred Reporting Items for Systematic Reviews and Meta-Analysis.
STROBE: Strengthening the Reporting of Observational Studies in Epidemiology for cross-sectional study
